# Controlling glycolysis to generate characteristic volatile organic compounds of lung cancer cells

**DOI:** 10.1038/s41598-024-67379-x

**Published:** 2024-07-17

**Authors:** Yajing Chu, Dianlong Ge, Jijuan Zhou, Yue Liu, Xiangxue Zheng, Wenting Liu, Li Ke, Yan Lu, Yannan Chu

**Affiliations:** 1 Anhui Province Key Laboratory of Medical Physics and Technology, Institute of Health and Medical Technology, Hefei Institutes of Physical Science, Chinese Academy of Sciences, Hefei, 230031 P. R. China; 2 University of Science and Technology of China, Hefei, 230026 P. R. China; 3 Hefei Cancer Hospital, Chinese Academy of Sciences, Hefei, 230031 P. R. China

**Keywords:** Lung cancer, Cytological examination, Glycolysis regulation, VOCs, SPME–GC–MS, Metabolomics, Diagnostic markers, Lung cancer, Analytical chemistry

## Abstract

Characteristic volatile organic compounds (VOCs) are anticipated to be used for the identification of lung cancer cells. However, to date, consistent biomarkers of VOCs in lung cancer cells have not been obtained through direct comparison between cancer and healthy groups. In this study, we regulated the glycolysis, a common metabolic process in cancer cells, and employed solid phase microextraction gas chromatography mass spectrometry (SPME–GC–MS) combined with untargeted analysis to identify the characteristic VOCs shared by cancer cells. The VOCs released by three types of lung cancer cells (A549, PC-9, NCI-H460) and one normal lung epithelial cell (BEAS-2B) were detected using SPME–GC–MS, both in their resting state and after treatment with glycolysis inhibitors (2-Deoxy-d-glucose, 2-DG/3-Bromopyruvic acid, 3-BrPA). Untargeted analysis methods were employed to compare the VOC profiles between each type of cancer cell and normal cells before and after glycolysis regulation. Our findings revealed that compared to normal cells, the three types of lung cancer cells exhibited three common differential VOCs in their resting state: ethyl propionate, acetoin, and 3-decen-5-one. Furthermore, under glycolysis control, a single common differential VOC—acetoin was identified. Notably, acetoin levels increased by 2.60–3.29-fold in all three lung cancer cell lines upon the application of glycolysis inhibitors while remaining relatively stable in normal cells. To further elucidate the formation mechanism of acetoin, we investigated its production by blocking glutaminolysis. This interdisciplinary approach combining metabolic biochemistry with MS analysis through interventional synthetic VOCs holds great potential for revolutionizing the identification of lung cancer cells and paving the way for novel cytological examination techniques.

## Introduction

According to recent global cancer statistics, the number of new cases and deaths of lung cancer worldwide in 2020 were 2.20 and 1.79 million respectively, the incidence and mortality ranking second and first among all cancer types^1^. Tissue biopsy followed by cytological examination currently serves as the gold standard for clinical diagnosis of lung cancer^2–4^. However, it is important to acknowledge that temporal and spatial heterogeneity in the morphology and structure of lung cancer cells may impact diagnostic reliability^5^. Therefore, it is crucial to develop innovative cytological examination to aid in accurate clinical pathological diagnosis. Recent studies have demonstrated that diseased cells exhibit metabolic abnormalities leading to the production of unique odours known as volatile organic compounds (VOCs)^6^. These VOCs, even present in minute quantities, hold great potential in distinguishing cancer cells by reflecting their distinctive metabolic features^7^.

The identification of characteristic VOCs emitted by lung cancer cells in vitro have been a challenging task, plagued with difficulties. To date, experimental studies have yielded inconsistent results regarding the specific VOC profiles of lung cancer cells^8^. For instance, Jia et al. employed solid phase microextraction-gas chromatography-mass spectrometry (SPME–GC–MS) technique to investigate the volatiles released by non-small cell lung cancer (NSCLC), and observed higher levels of 2-butanol and acetone in large cell lung cancer compared to adenocarcinoma or squamous cell carcinoma^9^. Furthermore, variations were also noted among different cell lines within the same histological type. In two squamous cell carcinoma cell lines, H520 cells exhibited increased propanal emission while consuming hexanal; however, no significant changes were observed in either substance within the headspace of H226 cells. Similarly, when comparing two adenocarcinoma cell lines A549 and HCC827, A549 released more benzaldehyde whereas HCC827 showed higher toluene emissions^9^.

Different lung cancer cells produce distinct VOCs, which can be attributed to variations in genetic mutations among different cell lines^10^. For instance, Peled et al. employed the SPME–GC–MS technique and identified benzaldehyde, triethylamine/decanal, decanal and triethylamine as characteristic VOCs for V-Ki-ras2 Kirsten rat sarcoma viral oncogene homolog (KRAS) mutation, epidermal growth factor receptor (EGFR) mutation, fusion of the EML4 gene to the ALK gene (EML4-ALK) mutation and non-mutant groups respectively^11^. Furthermore, Davies et al. using the thermal desorption-gas chromatography-mass spectrometry (TD-GC–MS) technique observed an increase in 2-methyl-pentane while a decrease in 2,2,3-trimethyl-pentane emissions from the human bronchial epithelial cell line 3KT upon TP53 gene knockout^12^.

Despite the presence of diverse gene mutations in lung cancer cells, these cells predominantly rely on glycolysis, a common biochemical process known as the Warburg effect, for their energy supply^13^. Exploiting this phenomenon, positron emission tomography-computed tomography (PET-CT) has been employed for clinical cancer diagnosis^14^. Therefore, this study proposes a novel approach to induce the production of VOCs with common features in lung cancer cells by regulating the glycolysis process. To this end, three types of lung cancer cells (A549, PC-9, NCI-H460) and one normal lung epithelial cell (BEAS-2B) were selected. SPME–GC–MS technique was used to analyze the VOCs released by these cells in both resting and glycolysis inhibitor-treated states (2-Deoxy-d-glucose, 2-DG/3-Bromopyruvic acid, 3-BrPA). Furthermore, potential biochemical sources of characteristic VOCs were investigated. The experimental process and schematic diagram are presented in Fig. S1. This study aims to induce specific VOCs production in lung cancer cells through manipulation of the glycolysis process with the objective of utilizing these VOCs as the potential biomarker for distinguishing cancer cells from normal cells in vitro. This approach may provide a novel method for developing cytological examination techniques.

## Materials and methods

### Cell lines

The lung cancer cell lines A549, PC-9, NCI-H460, and the normal lung epithelial cell line BEAS-2B used in this experiment were purchased from the American Type Culture Collection (ATCC) and cryopreserved in our laboratory’s liquid nitrogen tank at a temperature of − 192 °C. All cells underwent Short Tandem Repeat (STR) identification and mycoplasma detection.

### Chemical reagents

2-Deoxy-d-glucose (2-DG, CAS. No.: 154-17-6, Cat. No.: HY-13966, purity ≥ 98%), 3-Bromopyruvic acid (3-BrPA, CAS. No.: 1113-59-3, Cat. No.: HY-19992, purity ≥ 98%), and phenyl acetate (PA, CAS. No.: 122-79-2, Cat. No: HY-128733, purity: 99.02%) were obtained from Med Chem Express USA. Ethanol (CAS. No.: 64-17-5), acetone (CAS. No.: 67-64-1), trichloromethane (CAS. No.: 67-66-3), 1-butanol (CAS. No.: 71-36-3), acetoin (CAS. No.: 513-86-0), 3-methyl-1-butanol (CAS.No.: 123-51-3), ethylbeneze (CAS. No.: 100-41-4), p-xylene (CAS. No.: 106-42-3), 2-ethyl-1-hexanol (CAS. No.: 50373-29-0) et al*.* were purchased from Shanghai Aladdin Biochemical Technology Co., Ltd. The culture medium RPMI-1640, phosphate buffered solution (PBS) and fetal bovine serum (FBS) were provided by Biological Industries Israel Beit Haemek Ltd. Pancreatic enzyme and penicillin–streptomycin was obtained from Beyotime Biotech. Inc. Cell Counting kit-8 (CCK8) was offered by Biosharp Biotechnology Ltd.

### Cell culture

A549, PC-9, NCI-H460, and BEAS-2B cells were cultured in RPMI-1640 medium supplemented with 10% FBS and 1% penicillin/streptomycin at 37 °C under a 5% CO_2_ atmosphere in an incubator. The culture medium was refreshed every other day. To minimize the potential influence of plastic products on cell volatiles, the cells were transferred to T-25 cm^3^ glass culture flask^15^ at the logarithmic growth stage. Each glass flask was inoculated with 8 × 10^5^ cells, and the fresh culture medium containing different concentrations of inhibitors was replaced the next day. 3–6 parallel samples were set for each group of experiments. To prevent cross-contamination of VOCs released by different cell types within the incubator, parafilm membrane (p-m996; Bemis, USA) was immediately applied after completing the aforementioned procedures. The performance of parafilm membrane has been validated in our previous studies and shown not to interfere with our experimental results^15^. After culturing for 24 h at 37 °C under a 5% CO_2_ atmosphere in an incubator, detection was performed using SPME–GC–MS.

### Cell viability evaluation

The cell viability was assessed using the CCK8 assay according to the manufacturer’s instructions in order to determine the optimal concentration of inhibitors. Take 2-DG as an example, cells were seeded at a density of 5 × 10^3^ cells per well in a 96-well plate and incubated for 24 h. Subsequently, the cells were treated with different final concentrations (0, 5, 10, 20, 30, 40 mmol/L) of 2-DG for another 24 h. After removing the supernatant, each well was supplemented with 10 μL of CCK8 reagent. Following a further incubation period of 1.5 h with CCK8 reagent, absorbance at a wavelength of 450 nm was measured using Varioskan Flash microplate reader (Thermo Fisher Scientific, Rockford, IL, USA). The results are presented as percentage (%) cell viability relative to control conditions. The optimum concentration of 3-BrPA was determined using the same methodology. Low concentrations of PA that did not affect cell viability were employed in this study.

### SPME process and GC–MS condition

SPME, as a pre-treatment method for the extraction of volatiles, enables the combination of sampling and preconcentration in a single step, thereby simplifying the extraction process^16,17^. The selection of SPME fiber type, extraction time, and extraction temperature are shown in Figs. S2, S3. Similar with our previous research, we selected an SPME fiber (65 μm PDMS/DVB) based on its ability to enrich VOCs and extraction efficiency^15,18^. Prior to each SPME procedure, the extracted fiber was subjected to a 30-min pre-treatment at the 200 °C injection port of GC–MS to eliminate potential residual substances that could interfere with subsequent analysis of cell volatiles. Subsequently, the SPME fiber was introduced into the headspace of the cell culture flask for VOCs extraction at 37 °C incubator for 20 min. Throughout the experiment, only authorized personnel were allowed access to maintain air composition stability within the laboratory environment.

SPME–GC–MS detection and analysis were carried out on the gas chromatography triple quadrupole mass spectrometer (TSQ Quantum XLS, Thermo Fisher, USA). The column had a length of 30 m, an inner diameter of 0.32 mm, and a film thickness of 1.8 μm (Thermo, Bellefonte, USA), with the fixed-phase materials consisting of 6% cyanopropylphenyl and 94% polysiloxane. Initially, the column temperature was set at 50 °C for 0.5 min before being increased at a rate of 10 °C/min to reach 180 °C; it was then maintained for an additional 2 min before further elevation to 200 °C at a rate of 15 °C/min and subsequent maintenance for another 5 min. The flow rate of carrier gas helium (99.999%) was 1.5 mL/min with splitless injection mode. The MS conditions were consistent with our previous work^15^: electron impact energy set at 70 eV; mass scanning range from 45 to 200 amu; and utilization of the first quadruple rod as ion filter. Perfluorotributamine, MASS Grade (SynQuest Laboratories USA) was utilized as a corrective fluid to calibrate mass spectrometer. The sensitivity was checked prior to each test and its consistent performance is depicted in Fig. S4a. The relative intensity fluctuation is 7.39%, which proves the stability of the instrument.

### Statistical analysis

After GC–MS detection, the Xcalibur 2.2 (Thermo, USA) software was utilized to export all sample chromatographic peak areas. The selected chromatographic peaks were confirmed by NIST database to correct the shift of chromatographic peaks caused by different experiments. Chromatographic peaks with areas exceeding 1 × 10^5^ were selected and labeled as VOC1–VOC135 in ascending order of their retention time. Subsequently, orthogonal partial least squares discriminant analysis (OPLS-DA), the Mann–Whitney *U* test, and fold change (FC: ratio of lung cancer cell chromatographic peak area value to normal cell chromatographic peak value) were applied to analyze the 135 chromatographic peak areas of three types of lung cancer cells and one normal cell under resting state and glycolysis inhibition conditions. A substance corresponding to a chromatographic peak was considered significantly variable if it met the following criteria: variable importance projection (VIP) > 1, p-value < 0.01 (the p-value of the Mann–Whitney *U* test, p < 0.01, Benjamini–Hochberg corrected), and FC > 2. The qualitative analysis proceeded as follows: initially, potential substance candidates were selected based on a Reverse Search Index (RSI) value exceeding 800 from the NIST database. Subsequently, the retention index (RI) for certain VOC was determined using the experimental retention times. By comparing these RI values with those found in Refs.^19–21^, the most probable VOCs were ultimately identified from the substance candidates provided by the NIST database. Finally, a portion of the VOCs were definitively identified through the use of chemical standards. Table S1 shows the specific information and qualitative parameters of differential VOCs. The OPLS-DA analysis was carried out using SIMCA P14.1 while the Mann–Whitney *U* test was performed using IBM SPSS Statistics 25.

## Results and discussion

### Determination of the concentration range of inhibitors

**Figure 1:**
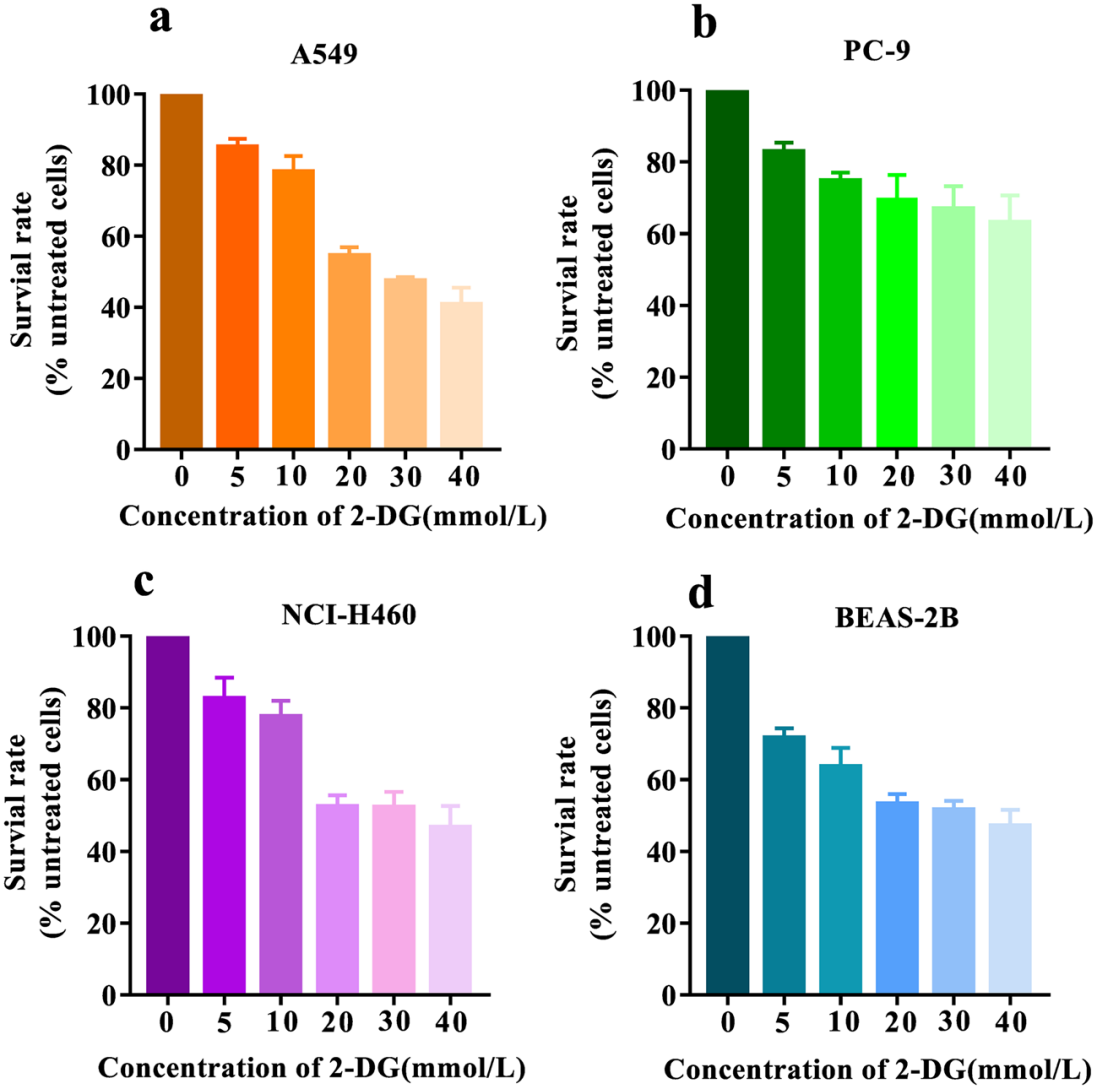
Survival rate of four cell types (A549, PC-9, NCI-H460, BEAS-2B) were assessed under varying concentrations of 2-DG.

In order to determine the appropriate concentration range of a glycolysis inhibitor, we investigated the relationship between the concentration of 2-DG and cell survival. The experimental results are presented in Fig. 1. Following treatment with varying concentrations of 2-DG, the survival rate of four different cell types (A549, PC-9, NCI-H460, BEAS-2B) exhibited a dose-dependent decrease with increasing inhibitor concentration. Specifically, as the concentration of 2-DG increased from 0 to 40 mmol/L, the survival rate decreased from 100 to 40% for A549 cells, from 100 to 63% for PC-9 cells, from 100 to 47% for NCI-H460 cells, and from 100 to 47% for BEAS-2B cells. Notably, BEAS-2B cells demonstrated higher sensitivity towards low concentrations of 2-DG compared to other cell types; conversely, A549 cells displayed greater sensitivity towards high concentrations of 2-DG.

To ensure that the characteristic VOCs are from living cells, according to the principle that the cell survival rate is nearly 80%, we finally chose the concentration of the experimental inhibitor as 2-DG of 10 mmol/L.

### Differences in VOCs between lung cancer cells and normal cells in resting state

**Figure 2:**
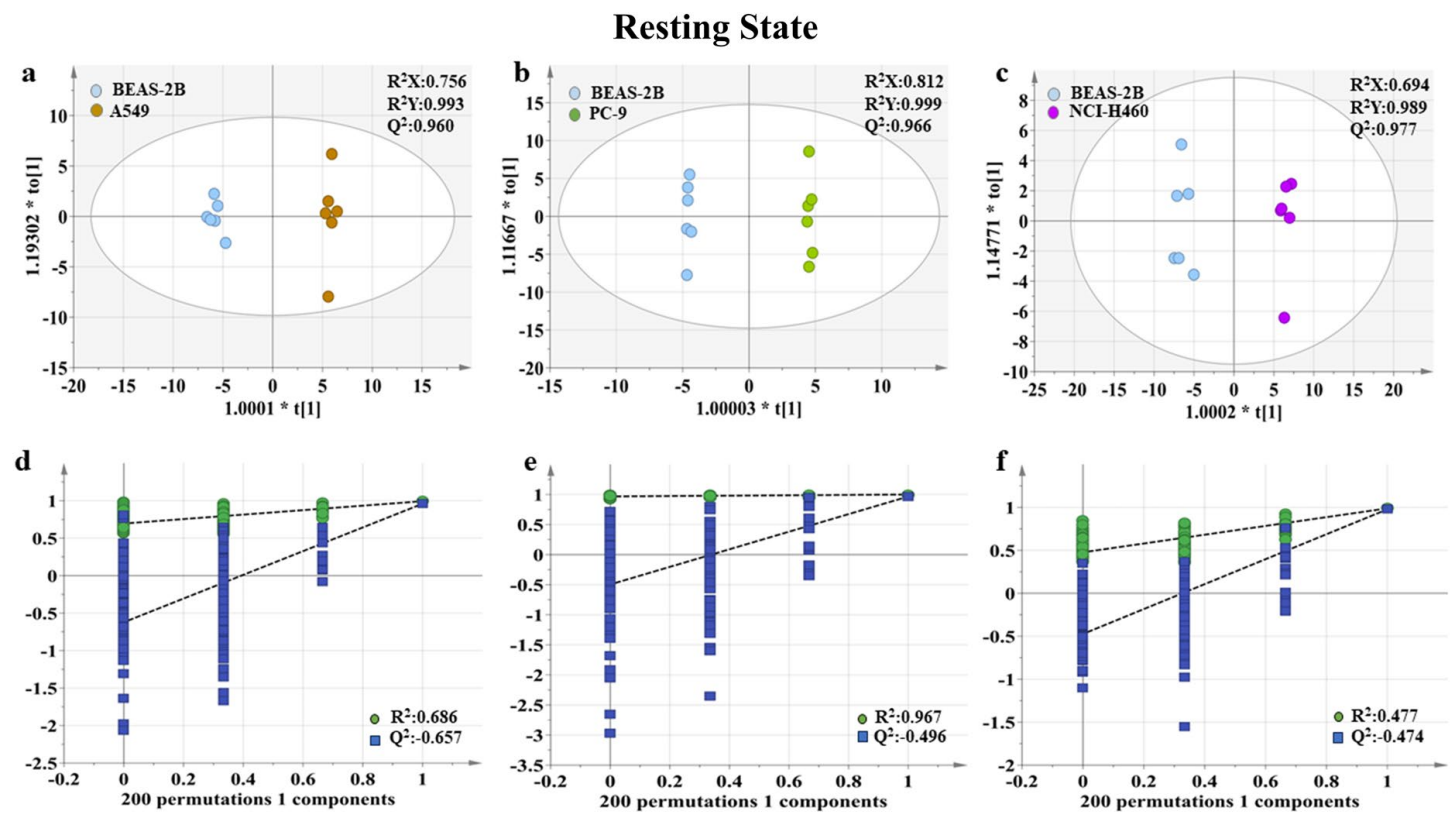
A549 (**a**)/PC-9 (**b**)/NCI-H460 (**c**) and BEAS-2B cells headspace VOCs OPLS-DA score plots in resting state (**a**–**c**), and the results of their corresponding 200 permutation tests (**d**–**f**).

In the resting state, the headspace VOCs of A549, PC-9, NCI-H460, and BEAS-2B were analyzed using the SPME–GC–MS technique. OPLS-DA was conducted utilizing the VOCs released by three distinct types of lung cancer cells, as well as normal cells during resting state. The resulting score plots are presented in Fig. 2a–c, where it is evident that BEAS-2B cells exhibit a clear separation from the three lung cancer cell types. Notably, these separations exhibit high goodness of fitting (R^2^Y) and prediction (Q^2^), indicating the robustness of the model. To further validate the efficacy of the OPLS-DA approach, 200 permutation tests were performed, and the results are depicted in Fig. 2d–f. The R^2^ and Q^2^ values obtained from the actual model exceed all corresponding values in the permutation models, unequivocally demonstrating the validity and statistical significance of the OPLS-DA analysis conducted in this study.

Following OPLS-DA, Mann–Whitney *U* test, and FC analysis, the results revealed distinct VOC profiles between each type of lung cancer cell and normal cells. Initially, based on the *U*-test analysis and FC a Volcano plot (Fig. S5a–c) was generated to visualize the changes in VOCs between each lung cell lines and normal cell line during the resting state. Subsequently, with reference to the OPLS-DA VIP > 1 and NIST database RSI > 800 differential VOCs were summarized in Table 1. As depicted in Venn diagram of difference VOCs (Fig. S6a), three common substances were found: VOC19 (ethyl propionate), VOC23 (acetoin), and VOC80 (3-decen-5-one). Ethyl propionate and 3-decen-5-one exhibited lower levels in the headspace of each lung cancer cell type compared to normal cells; conversely, acetoin showed higher levels. In conclusion, these three aforementioned VOCs may serve as potential biomarkers for distinguishing lung cancer cells from normal cells.

**Table 1:** Differences in VOCs between lung cancer cells (A549, PC-9, NCI-H460) and normal cells (BEAS-2B) in resting state.

Differential VOCs	Number	Name	HMDB ID	CAS	RT (min)	VIP	FDR	Log_2_(FC)
A549 vs BEAS-2B								
1	VOC4	Acetone	HMDB0001659	67-64-1	2.01	1.40	< 0.01	− 1.46
2	VOC9	3,3,4,4-Tetrafluoro-hexane	–	648-36-2	3.12	1.36	< 0.01	− 1.66
3	VOC14	Heptane	HMDB0031447	142-82-5	3.93	1.37	< 0.01	− 3.23
4	VOC15	1-Butanol	HMDB0004327	71-36-3	4.08	1.38	< 0.01	− 2.72
5	VOC19	Ethyl propionate	HMDB0030058	105-37-3	4.45	1.33	< 0.01	− 1.61
6	VOC22	2-Methyl-heptane	–	592-27-8	4.98	1.33	< 0.01	− 3.41
7	VOC23	Acetoin	HMDB0003243	513-86-0	5.14	1.36	< 0.01	2.50
8	VOC28	2,4-Dimethyl-heptane	–	2213-23-2	5.96	1.35	< 0.01	− 3.78
9	VOC31	2,4-Dimethyl-1-heptene	–	NA	6.39	1.33	< 0.01	− 1.43
10	VOC32	3-Nonene	–	20063-77-8	6.54	1.31	< 0.01	− 4.12
11	VOC33	4-Methyl-octane	–	2216-34-4	6.70	1.32	< 0.01	− 2.66
12	VOC37	p-Xylene	HMDB0059924	106-42-3	7.24	1.28	< 0.01	− 1.08
13	VOC47	2-Methyl-nonane	–	871-83-0	8.46	1.24	< 0.01	− 1.67
14	VOC49	Oxime-, methoxy-phenyl-	–	NA	9.00	1.38	< 0.01	− 2.42
15	VOC52	2-5-Dimethyl-nonane	–	17302-27-1	9.33	1.39	< 0.01	− 2.64
16	VOC54	4-Ethyl-2,2,6,6-tetramethy1-heptane	–	62108-31-0	9.51	1.40	< 0.01	− 1.74
17	VOC56	2,3,6,7-Tetramethyl-octane	–	52670-34-5	9.83	1.29	< 0.01	− 1.86
18	VOC60	2,2,4,4-Tetramethyl-petane	–	1070-87-7	10.24	1.27	< 0.01	− 1.31
19	VOC61	2-Ethyl-1-hexanol	HMDB0031231	NA	10.33	1.41	< 0.01	− 4.95
20	VOC78	Dodecane	HMDB0031444	112-40-3	12.36	1.38	< 0.01	− 1.58
21	VOC80	3-Decen-5-one	–	32064-73-6	12.76	1.26	< 0.01	− 1.12
22	VOC97	3,7-Dimethyl-undecane	–	17301-29-0	14.33	1.31	< 0.01	− 1.46
PC-9 vs BEAS-2B								
1	VOC3	Ethanol	HMDB0000108	64-17-5	1.78	1.52	< 0.01	− 1.64
2	VOC17	5,5-Dimethyl-(Z)-2-hexene	–	39761-61-0	4.23	1.71	< 0.01	− 1.47
3	VOC19	Ethyl propionate	HMDB0030058	105-37-3	4.45	1.53	< 0.01	− 1.09
4	VOC23	Acetoin	HMDB0003243	513-86-0	5.14	1.76	< 0.01	2.65
5	VOC55	2,2,7,7-Tetramethyl-octane	–	1071-31-4	9.71	1.62	< 0.01	1.02
6	VOC80	3-Decen-5-one	–	32064-73-6	12.76	1.94	< 0.01	− 1.35
NCI-H460 vs BEAS-2B								
1	VOC4	Acetone	HMDB0001659	67-64-1	2.01	1.31	< 0.01	− 1.48
2	VOC15	1-Butanol	HMDB0004327	71-36-3	4.08	1.28	< 0.01	− 1.16
3	VOC19	Ethyl propionate	HMDB0030058	105-37-3	4.45	1.25	< 0.01	− 1.81
4	VOC23	Acetoin	HMDB0003243	513-86-0	5.14	1.27	< 0.01	3.02
5	VOC28	2,4-Dimethyl-heptane	–	2213-23-2	5.96	1.26	< 0.01	− 4.99
6	VOC31	2,4-Dimethyl-1-heptene	–	NA	6.39	1.29	< 0.01	− 3.26
7	VOC32	3-Nonene	–	20063-77-8	6.54	1.23	< 0.01	− 6.76
8	VOC33	4-Methyl-octane	–	2216-34-4	6.70	1.24	< 0.01	− 3.51
9	VOC34	2-Methyl-ethyl ester butanoic acid	HMDB0033745	7452-79-1	6.85	1.28	< 0.01	− 2.74
10	VOC36	Ethylbeneze	HMDB0059905	100-41-4	7.12	1.26	< 0.01	− 1.03
11	VOC37	p-Xylene	HMDB0059924	106-42-3	7.24	1.30	< 0.01	− 2.09
12	VOC44	3-Carene	HMDB0035619	13466-78-9	8.23	1.28	< 0.01	− 1.83
13	VOC49	Oxime-, methoxy-phenyl-	–	NA	9.00	1.29	< 0.01	− 2.52
14	VOC50	Decane	HMDB0031450	124-18-5	9.07	1.29	< 0.01	− 3.26
15	VOC52	2-5-Dimethyl-nonane	–	17302-27-1	9.33	1.30	< 0.01	− 3.04
16	VOC61	2-Ethyl-1-hexanol	HMDB0031231	NA	10.33	1.31	< 0.01	− 3.22
17	VOC73	Dimethyl-benzenemethanol	–	617-94-7	11.65	1.31	< 0.01	− 1.07
18	VOC78	Dodecane	HMDB0031444	112-40-3	12.36	1.29	< 0.01	− 1.17
19	VOC80	3-Decen-5-one	–	32064-73-6	12.76	1.29	< 0.01	− 2.70
20	VOC82	2,6,10-Trimethyl-tetradecane	–	14905-56-7	12.98	1.04	< 0.01	2.73
21	VOC97	3,7-Dimethyl-undecane	–	17301-29-0	14.33	1.23	< 0.01	− 1.72
22	VOC119	Pentadecane	HMDB0059886	629-62-9	17.45	1.26	< 0.01	2.78
23	VOC133	Hexadecane	HMDB0033792	544-76-3	19.49	1.28	< 0.01	3.07

### Differences in VOCs between lung cancer cells and normal cells after glycolysis inhibition

**Figure 3:**
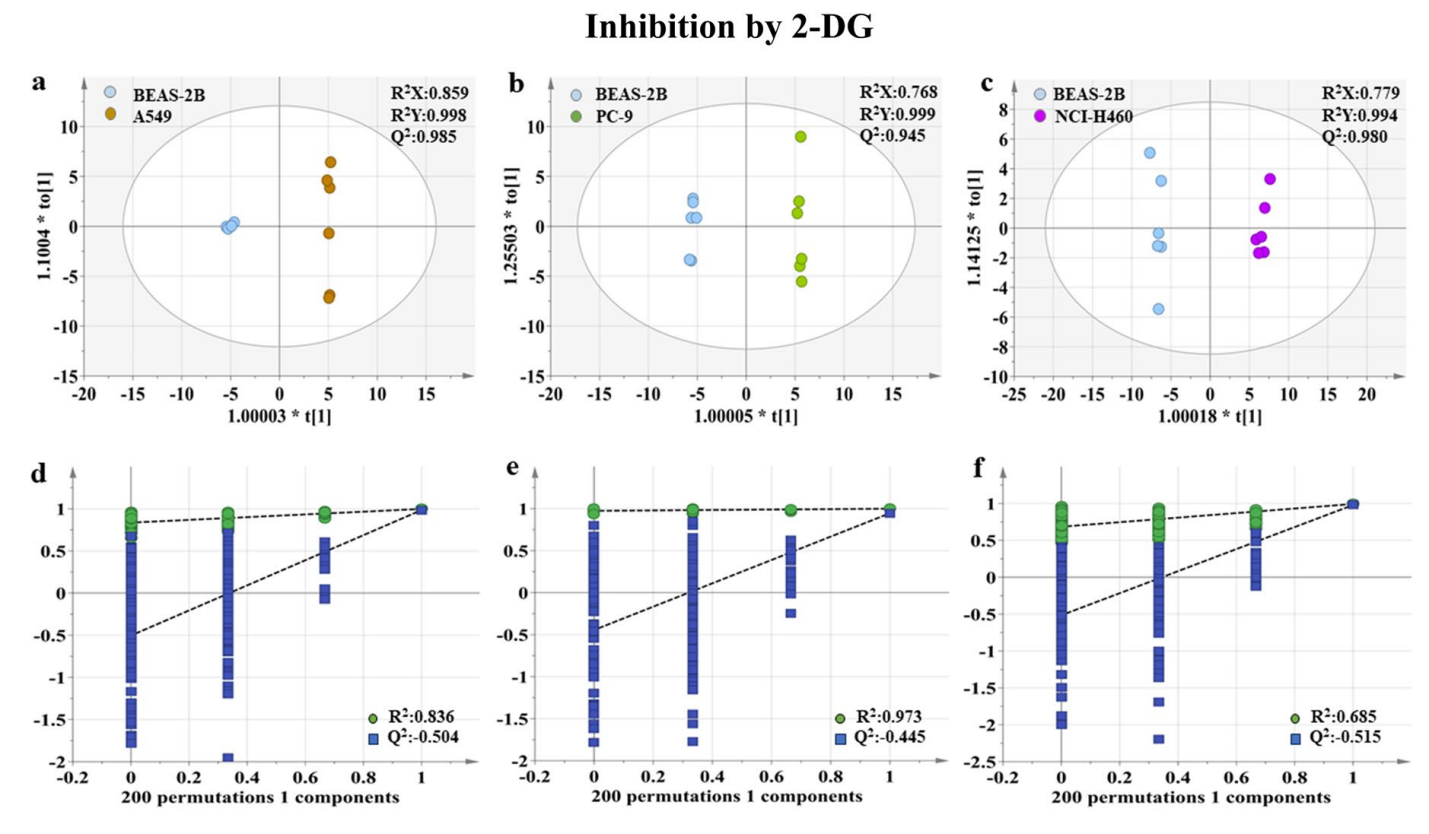
A549 (**a**)/PC-9 (**b**)/NCI-H460 (**c**) and BEAS-2B cells headspace VOCs OPLS-DA score plots after the inhibition by 2-DG (**a**–**c**), and the results of their corresponding 200 permutation tests (**d**–**f**).

After the glycolysis inhibition by 2-DG, headspace VOCs of A549, PC-9, NCI-H460, and BEAS-2B cells were analyzed using SPME–GC–MS technique and subjected to statistical analysis. OPLS-DA scores plots and corresponding 200 permutation tests are presented in Fig. 3. All three OPLS-DA models have high goodness of fitting and goodness of prediction. Additionally, the simulated values of 200 replacement tests of the corresponding models are greater than all their original values, indicating the effectiveness of the OPLS-DA model.

The Volcano plot (Fig. S5d–f) serves as a visualization tool to illustrate the alterations in VOCs between each lung cancer cell and normal cells after 2-DG regulation. Table 2 presents the differential VOCs information. Furthermore, the Venn diagram (Fig. S6b) provides a concise representation of the overlap among the three sets of differential VOCs, revealing that only one substance, VOC23 (acetoin), was consistently detected across all three groups. All three types of lung cancer cells exhibited higher levels of acetoin in the headspace compared to normal cells.

**Table 2:** Differences in VOCs between lung cancer cells (A549, PC-9, NCI-H460) and normal cells (BEAS-2B) after the glycolysis inhibition by 2-DG.

Differential VOCs	Number	Name	HMDB ID	CAS	RT (min)	VIP	FDR	Log_2_(FC)
A549 vs BEAS-2B								
1	VOC3	Ethanol	HMDB0000108	64-17-5	1.78	1.29	< 0.01	− 2.01
2	VOC9	3,3,4,4-Tetrafluoro-hexane	–	648-36-2	3.12	1.32	< 0.01	− 1.19
3	VOC23	Acetoin	HMDB0003243	513-86-0	5.14	1.36	< 0.01	3.69
4	VOC29	Pyrrole	HMDB0035924	109-97-7	6.07	1.40	< 0.01	1.36
5	VOC32	3-Nonene	–	20063-77-8	6.54	1.19	< 0.01	− 1.60
6	VOC49	Oxime-, methoxy-phenyl-	–	NA	9.00	1.43	< 0.01	− 1.52
7	VOC52	2-5-Dimethyl-nonane	–	17302-27-1	9.33	1.25	< 0.01	− 1.26
8	VOC54	4-Ethyl-2,2,6,6-tetramethy-heptane	–	62108-31-0	9.51	1.32	< 0.01	− 1.04
9	VOC61	2-Ethyl-1-hexanol	HMDB0031231	NA	10.33	1.49	< 0.01	− 4.50
10	VOC78	Dodecane	HMDB0031444	112-40-3	12.36	1.43	< 0.01	− 1.30
11	VOC97	3,7-Dimethyl-undecane	–	17301-29-0	14.33	1.42	< 0.01	− 1.59
PC-9 vs BEAS-2B								
1	VOC3	Ethanol	HMDB0000108	64-17-5	1.78	1.25	< 0.01	− 1.05
2	VOC13	3-Methyl-butanal	–	590-86-3	3.82	1.47	< 0.01	− 2.40
3	VOC15	1-Butanol	HMDB0004327	71-36-3	4.08	1.61	< 0.01	− 1.78
4	VOC17	5,5-Dimethyl-,(Z)-2-hexene	–	39761-61-0	4.23	1.41	< 0.01	− 2.17
5	VOC23	Acetoin	HMDB0003243	513-86-0	5.14	1.49	< 0.01	4.23
6	VOC44	3-Carene	HMDB0035619	13466-78-9	8.23	1.38	< 0.01	1.76
7	VOC80	3-Decen-5-one	–	32064-73-6	12.76	1.62	< 0.01	− 1.76
NCI-H460 vs BEAS-2B								
1	VOC13	3-Methyl-butanal	–	590-86-3	3.82	1.12	< 0.01	− 3.50
2	VOC15	1-Butanol	HMDB0004327	71-36-3	4.08	1.21	< 0.01	− 1.49
3	VOC23	Acetoin	HMDB0003243	513-86-0	5.14	1.12	< 0.01	4.36
4	VOC28	2,4-Dimethyl-heptane	–	2213-23-2	5.96	1.14	< 0.01	− 5.08
5	VOC31	2,4-Dimethyl-1-heptene	–	NA	6.39	1.17	< 0.01	− 3.68
6	VOC32	3-Nonene	–	20063-77-8	6.54	1.10	< 0.01	− 5.80
7	VOC33	4-Methyl-octane	–	2216-34-4	6.70	1.11	< 0.01	− 3.56
8	VOC34	2-Methyl-ethyl ester butanoic acid	HMDB0033745	7452-79-1	6.85	1.16	< 0.01	− 2.43
9	VOC44	3-Carene	HMDB0035619	13466-78-9	8.23	1.03	< 0.01	− 1.78
10	VOC49	Oxime-, methoxy-phenyl-	–	NA	9.00	1.20	< 0.01	− 2.23
11	VOC50	Decane	HMDB0031450	124-18-5	9.07	1.18	< 0.01	− 2.87
12	VOC52	2-5-Dimethyl-nonane	–	17302-27-1	9.33	1.19	< 0.01	− 2.62
13	VOC54	4-Ethyl-2-2-6-6-tetramethy1-heptane	–	62108-31-0	9.51	1.12	< 0.01	− 1.20
14	VOC61	2-Ethyl-1-hexanol	HMDB0031231	NA	10.33	1.21	< 0.01	− 3.11
15	VOC78	Dodecane	HMDB0031444	112-40-3	12.36	1.14	< 0.01	− 1.01
16	VOC80	3-Decen-5-one	–	32064-73-6	12.76	1.21	< 0.01	− 3.89
17	VOC97	3,7-Dimethyl-undecane	–	17301-29-0	14.33	1.20	< 0.01	− 1.55
18	VOC99	2-Undecanone	HMDB0033713	112-12-9	14.60	1.18	< 0.01	6.50

### Identification of characteristic VOC for lung cancer cells under glycolysis regulation

**Figure 4:**
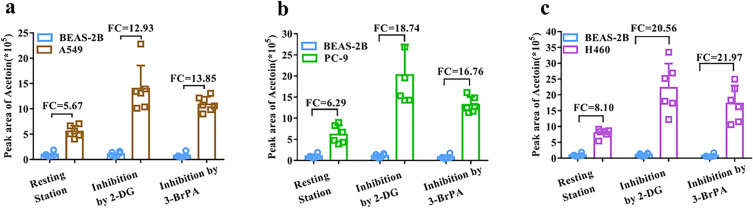
Differences in the peak area of acetoin in the headspace before and after glycolysis inhibition between lung cancer cells and normal cells.

It is worth emphasizing that in comparison to the resting state, the glycolysis inhibition by 2-DG significantly augmented the disparity in acetoin levels in the headspace of lung cancer cells (A549, PC-9, NCI-H460) and normal cells (BEAS-2B). The FC values increased from 5.67, 6.29, 8.10 times to 12.93, 18.74, and 20.56 times respectively (Fig. 4). To further validate the association between increased acetoin levels and glycolysis inhibition, we conducted a repetitive experiment using another glycolysis inhibitor (3-BrPA). Initially, an appropriate concentration range of 100 μmol/L was selected for 3-BrPA (Fig. S7). Subsequently, SPME–GC–MS detection and statistical analysis were performed on the headspace VOCs of A549, PC-9, NCI-H460, and BEAS-2B under glycolysis regulation with 3-BrPA treatment. Relevant results are presented in Fig. S8, Fig. S5h–j, Table S2, and Fig. S6c. Importantly, acetoin was still detected among all three groups of differential VOCs. The FC values for lung cancer cells (A549, PC-9, NCI-H460) and normal cells (BEAS-2B) increased from 5.67, 6.29, 8.10 times to 13.85, 16.76, 21.97 times, respectively (Fig. 4). Overall, glycolysis inhibition can significantly amplify the disparity in acetoin levels between lung cancer cells and normal cells. This suggests that acetoin could serve as the characteristic VOC for distinguishing between lung cancer cells and normal cells under glycolysis regulation.

We also noticed that Feinberg et al.^22^ mentioned that the substance with m/z of 49 experienced the most significant reduction in A549 cells after the action of inhibitor 3-BrPA, which speculated to be methanethiol. In our experiment, no characteristic VOC with m/z of 49 was detected, including methanethiol, and the reason for the two differences is not clear.

### Biochemical sources of characteristic VOC for lung cancer cells under glycolysis regulation

**Figure 5:**
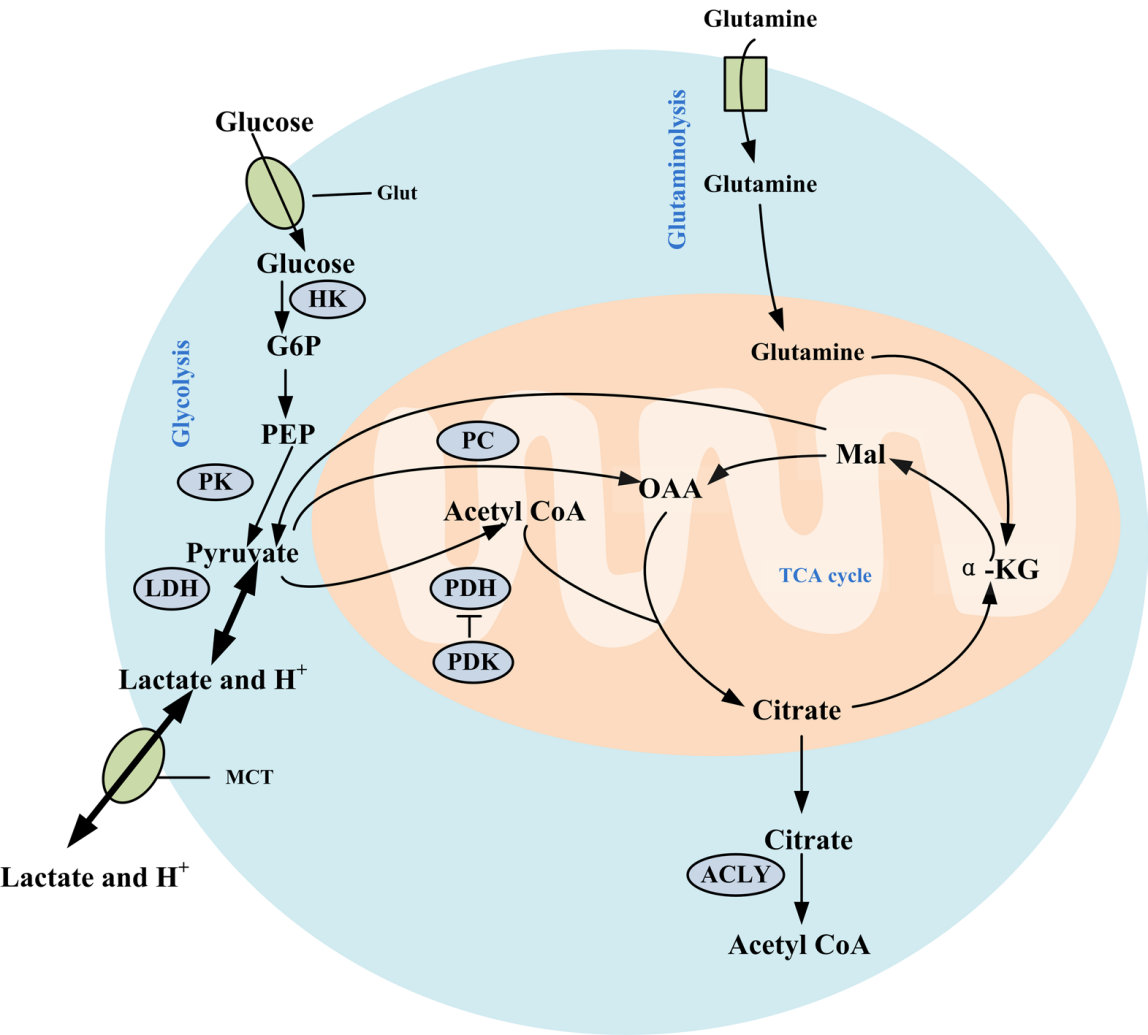
Metabolism pathway of glucose and glutamine in lung cancer cells. The bold arrow indicates that the process is dominant. *Glut* glucose transporter, *HK* hexokinase, *G6P* glucose-6-phosphate, *PEP* phosphoenol pyruvate, *PK* pyruvate kinase, *LDH* lactate dehydrogenase, *MCT* monocarboxylate transporter, *PC* pyruvate carboxylase, *PDH* pyruvate dehydrogenase, *PDK* pyruvate dehydrogenase kinase, *OAA* oxaloacetate, *Mal* malate, *α-KG* α-ketoglutarate, *ACLY* ATP citrate lyase, *TCA* tricarboxylic acid cycle.

Several studies have observed the presence of acetoin^23–25^ in the breath of individuals with lung cancer, Sanni et al.^25^ have identified that this compound may originated from the glucose metabolism of oral bacteria. To our knowledge, there is currently no available research on the biochemical origins of acetoin in human cells. However, it has been confirmed that three distinct pathways exist for the production of acetoin in bacteria and yeast, which are schematically represented in Fig. S9^26–28^. Pyruvate serves as the starting substrate to produce acetoin based on these three pathways. Glycolysis is a process by which glucose is broken down into pyruvate in the cytoplasm, releasing energy. When glycolysis is inhibited, mitochondria increase their utilization of glutamine to produce organic molecules such as pyruvate and acetyl CoA to maintain energy and biosynthesis needs^29^, as depicted in Fig. 5.

**Figure 6:**
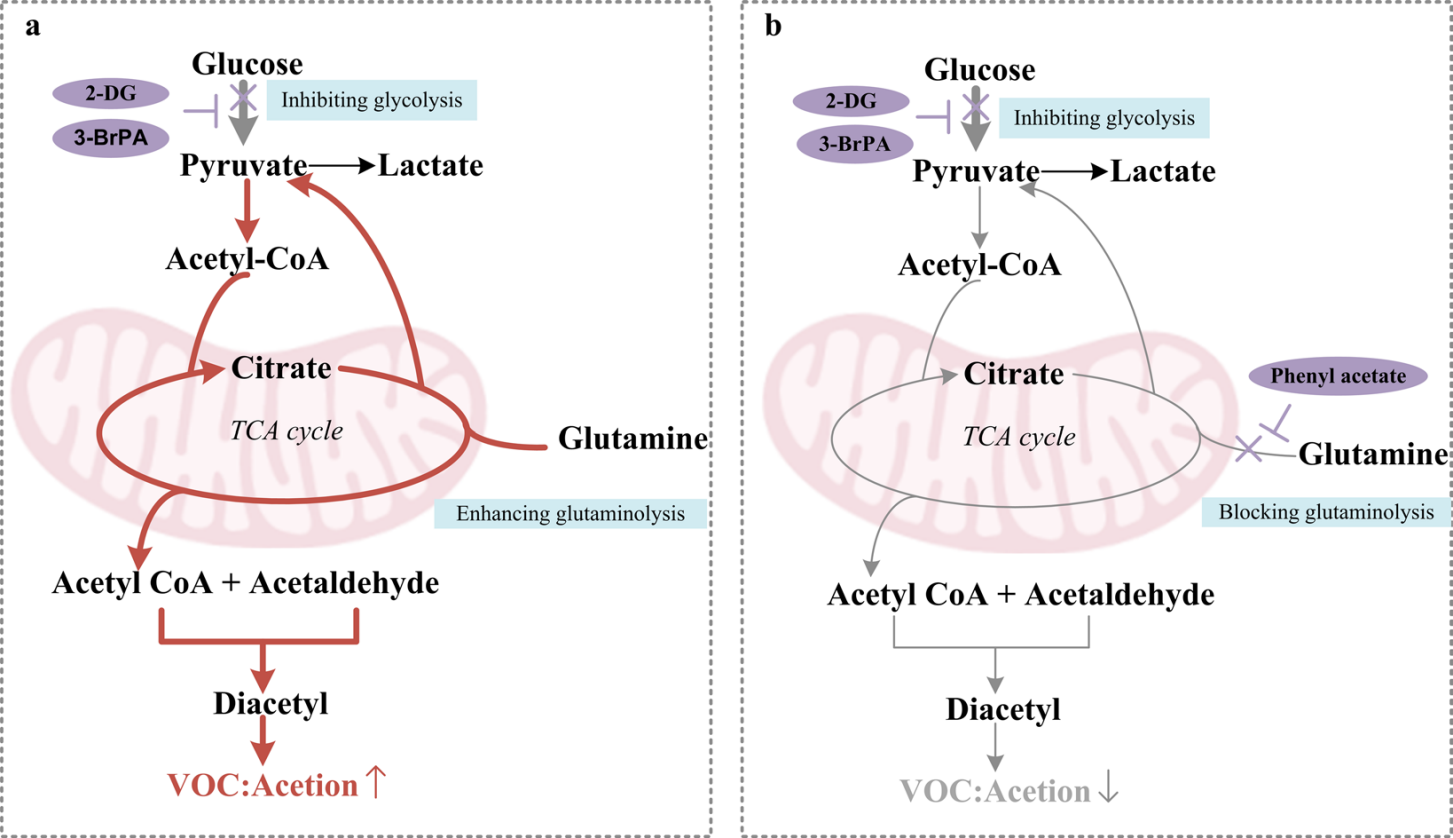
(**a**) Mechanism underlying the upregulation of acetoin resulting from glycolysis inhibition. (**b**) Mechanism explaining the downregulation of acetoin due to combined inhibition of glycolysis and blockade of glutaminolysis. The thickness of the arrows represents pathway strength or weakness.

Therefore, we hypothesize that the glycolysis inhibition leads to an upregulation of the glutamine degradation pathway, resulting in a compensatory increase in acetoin production, as illustrated in Fig. 6a. Consequently, it is reasonable to consider whether concurrent restriction of the glutamine breakdown pathway and glycolysis inhibition would lead to a reduction in acetoin (Fig. 6b).

**Figure 7:**
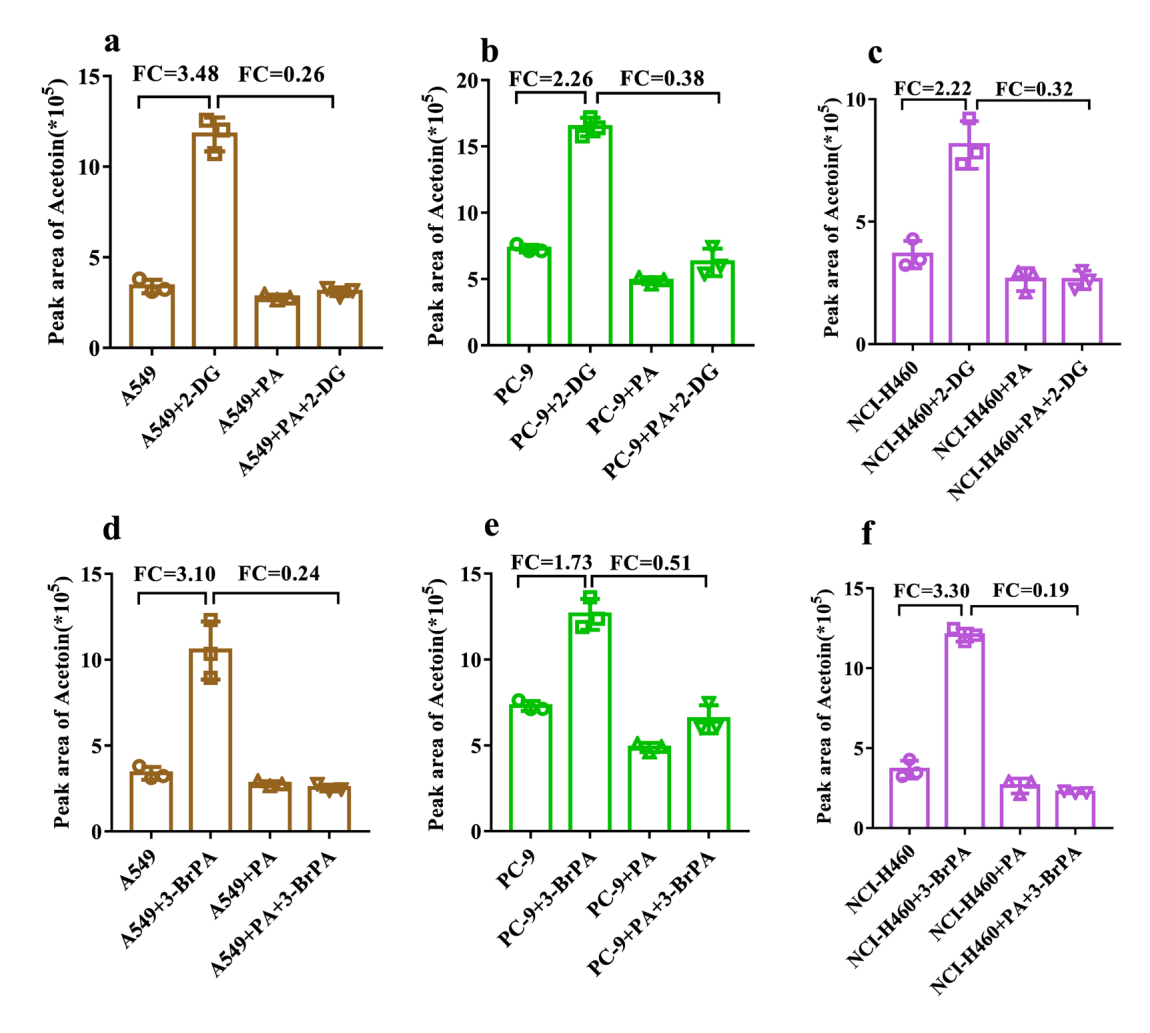
Alteration in the peak area of acetoin within the headspace of lung cancer cells when 2-DG/3-BrPA inhibiting glycolysis while PA blocking glutaminolysis.

To investigate the potential of glycolysis inhibition in enhancing glutaminolysis for pyruvate production in lung cancer cells, we used 2-DG/3-BrPA to inhibit glycolysis while utilizing PA to block glutaminolysis^30^. As depicted in Fig. 7, compared to the resting state, treatment with 2-DG/3-BrPA significantly increased acetoin levels in the headspace of all three types of lung cancer cells. However, there was no significant alteration observed when treated with PA alone. Moreover, concurrent administration of 2-DG/3-BrPA and PA effectively suppressed the upward trend of acetoin release. These findings further validate our hypothesis (Fig. 6b). It is evident that the elevation in acetoin is associated with compensatory enhancement of glutaminolysis.

## Conclusions

To address the challenge of VOC replication in lung cancer cells, we focus on glycolysis, a common metabolic process in cancer cells. Initially, we regulated the metabolic processes of lung cancer cells (A549, PC-9, NCI-H460) and normal lung epithelial cell (BEAS-2B) using the glycolysis inhibitors (2-DG and 3-BrPA). We then analyzed the cell headspace VOCs before and after glycolysis regulation using SPME–GC–MS technique combined with untargeted analysis methods. Our results showed that application of glycolysis inhibitors significantly increased acetoin release by all three types of lung cancer cells but not by normal lung epithelial cells. This observation further highlighted the disparity between lung cancer cells and normal cells under chemical intervention. Additionally, we investigated the mechanism of acetoin production by examining pharmacological effects of glycolysis inhibitors. Our interdisciplinary approach combining biochemical metabolism and mass spectrometry analysis can differentiate between lung cancer and normal cells through intervening in VOC synthesis. These findings may have significant implications for cytological examination and diagnosis.

While this study provides valuable insights into VOCs in lung cancer cells, further research is needed to understand variations in VOCs among cell lines with varying glycolysis levels and to assess its broader applicability. Future work will focus on co-culturing normal and lung cancer cells and detecting VOCs in human tumor tissues under controlled glycolysis conditions, with the goal of translating this research into clinical applications.

### Supplementary Information

Supplementary Information.

## Data Availability

Data that support the findings of this study have been deposited in the Science Data Bank with the link 10.57760/sciencedb.09849.
