# Supplementary material for: Controlling glycolysis to generate characteristic volatile organic compounds of lung cancer cells
Source: Sci Rep. 2024 Jul 17;14:16561. doi: 10.1038/s41598-024-67379-x (PMC11255210; doi:10.1038/s41598-024-67379-x)
Supplement: Supplementary file 1 — Supplementary Information. [file 41598_2024_67379_MOESM1_ESM.doc]

**Supplementary Information to:**

**Article Title:** Controlling glycolysis to generate characteristic volatile organic compounds of lung cancer cells

**Journal name**: Scientific Reports

Yajing Chu^1,2^, Dianlong Ge^1,3,*^, Jijuan Zhou^1,2^, Yue Liu^1,2^, Xiangxue Zheng^1^, Wenting Liu^1,2^, Li Ke^1,2^, Yan Lu^1,3,*^, Yannan Chu^1,3,*^

^1^Anhui Province Key Laboratory of Medical Physics and Technology, Institute of Health and Medical Technology, Hefei Institutes of Physical Science, Chinese Academy of Sciences, Hefei, 230031, P. R. China.

^2^University of Science and Technology of China, Hefei, 230026, China

^3^Hefei Cancer Hospital, Chinese Academy of Sciences, Hefei, 230031, P. R. China

*Corresponding. gedl@cmpt.ac.cn, ylu@cmpt.ac.cn, ychu@aiofm.ac.cn

**Table of Contents**

**Figure S1.** Schematic diagram of experimental principle and steps. 3

**Figure S2.** Optimization of SPME conditions. 5

**Figure S3.** (a) Cell morphology after detection at 37 °C; (b) Cell morphology after detection at 50 °C. 5

**Figure S4.** (a) Stability analysis of the GC-MS instrument showing the relative fluctuation of intensity; (b) Peak area corresponding to different concentrations of ethanol. 6

**Figure S5.** Volcano plots comparing the VOCs in headspace between Normal cell / Lung cancer cell, with significance based on p* < 0.01, FC < 0.5 or > 2. 7

**Figure S6.** The Venn diagram of difference VOCs between three lung cancer cells and normal cells 8

**Figure S7.** Survival rate of four cell types (A549, PC-9, NCI-H460, BEAS-2B) were assessed under varying concentrations of 3-BrPA. 9

**Figure S8.** BEAS-2B and A549 (a) / PC-9 (b) / NCI-H460 (c) cells headspace VOCs OPLS-DA score plots after the inhibition by 3-BrPA (a, b, c), and the results of their corresponding 200 permutation tests (d, e, f). 10

**Figure S9.** Pathway of acetoin production. 11

**Table S1.** Qualitative parameters of differential VOCs. 12

**Table S2.** Differences in VOCs between lung cancer cells (A549, PC-9, NCI-H460) and normal cells (BEAS-2B) after the glycolysis inhibition by 3-BrPA. 13


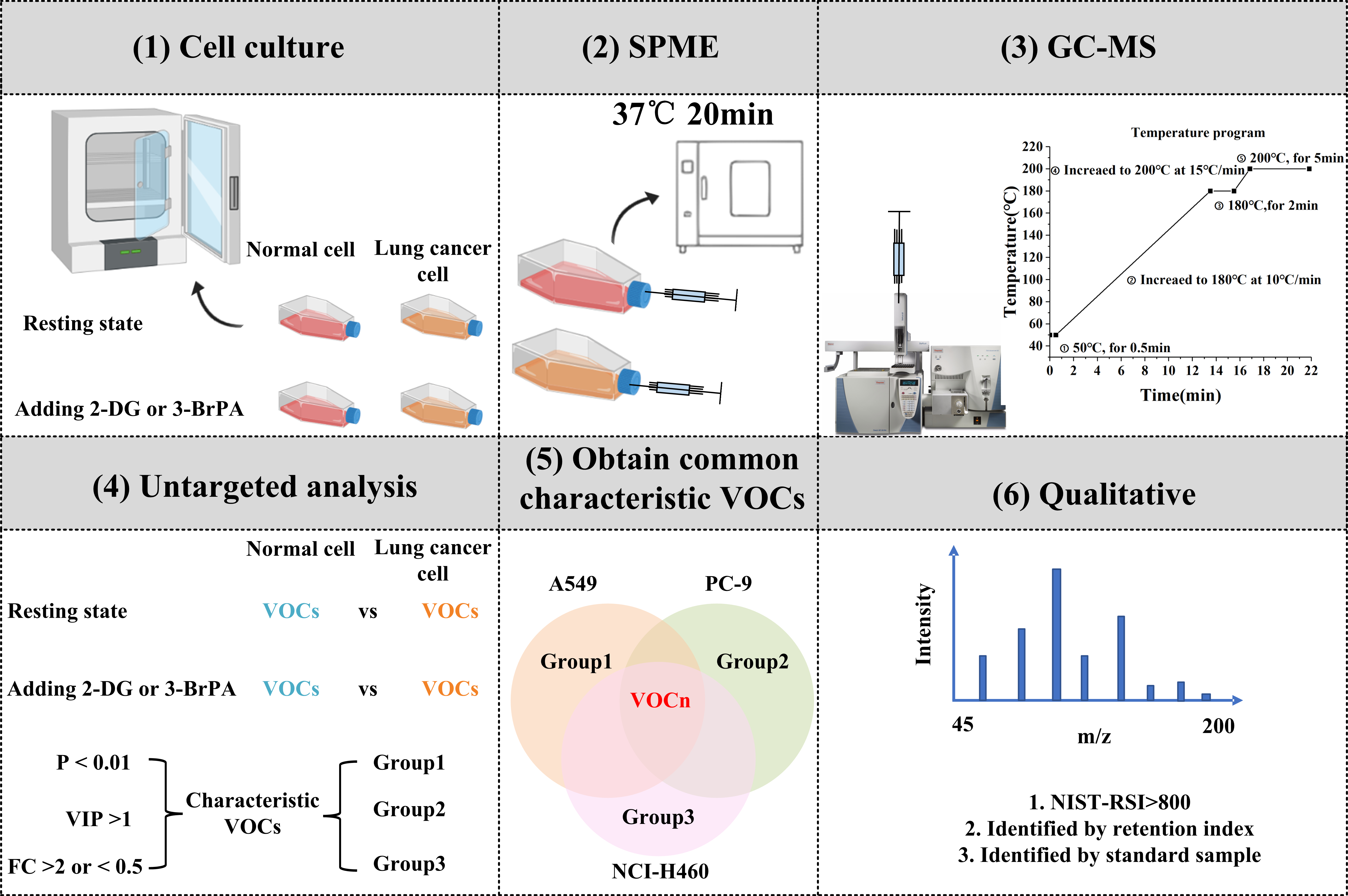


**Figure S1.** Schematic diagram of experimental principle and steps: (1) Cell culture and inhibitor treatment; (2) SPME procedure; (3) GC-MS detection and temperature programmed; (4) Untargeted statistical analysis acording Mann-Whitney U test, OPLS-DA and FC. p: p-value, VIP: variable importance projection, FC: fold change (ratio of lung cancer cell chromatographic peak area value to normal cell chromatographic peak value). (5) Differential VOCs of three kinds of lung cancer cells were compared to obtain the common characteristic VOCs of lung cancer cells. (6) Characteristic VOCs were qualitatively determined according to NIST database and standard chemicals. RSI: Reverse Search Index.

**Materials and methods**

The selection of SPME, extraction temperature, and extraction time.

(1) The selection of SPME fiber (65 μm PDMS/DVB)

We employed three distinct types of SPME fibers in our experiments: SPME-1 (50/30 μm DVB/CAR/PDMS), SPME-2 (65 μm PDMS/DVB), and SPME-3 (100 μm PDMS). Our analysis of the enrichment capacity of these fibers was conducted on the RPMI-1640 medium used in the experiment. As depicted in Fig. S2a, the enumeration of VOCs enriched by each SPME type revealed little variation among them. However, upon further scrutiny of the enrichment efficiency (Fig. S2b), we observed that SPME-2 displayed a higher sensitivity towards the target VOCs. Consequently, SPME-2 was selected for our subsequent investigations, owing to its superior performance. Notably, SPME-2 was also utilized in our previous study^1,2^, further validating its suitability for our current research.

(2) Selection of SPME times (20 min)

We tested extraction times of 10 min, 20 min, and 30 min, as depicted in Fig. S2c. Initially, when the enrichment duration was set at 10 min, we observed that the intensity of VOCs was significantly low. After conducting further analysis, we found that the intensities of VOCs enriched at 20 min and 30 min were remarkably similar. Balancing the timeliness of the experiment with the need for sufficient VOCs intensity, we ultimately opted for an extraction time of 20 min.

(3) Selection of SPME temperature (37 ℃)

During the pre-experimental phase, we selected the lung cancer cell line PC-9 for optimizing the extraction temperature. Given that the standard cell culture environment is maintained at 37 ℃, we initially favored this temperature for VOC extraction. Additionally, we conducted an enrichment detection of VOCs under varying heating conditions. As depicted in Fig. S2d, the peak intensity of VOCs was notably higher at 37 ℃ compared to 50 ℃. During our experiments, we observed that when the cells were heated to 50 ℃ for VOC extraction, the cellular structure underwent significant changes. Post-extraction microscopic analysis revealed irregular edges and distorted shapes, as illustrated in Fig. S3a-b. These findings indicated that the higher temperature was not suitable for preserving cell integrity and potentially affected the quality of VOC extraction. Therefore, based on the VOC enrichment efficiency and the preservation of cellular integrity, we ultimately opted for 37 ℃ as the optimal extraction temperature.


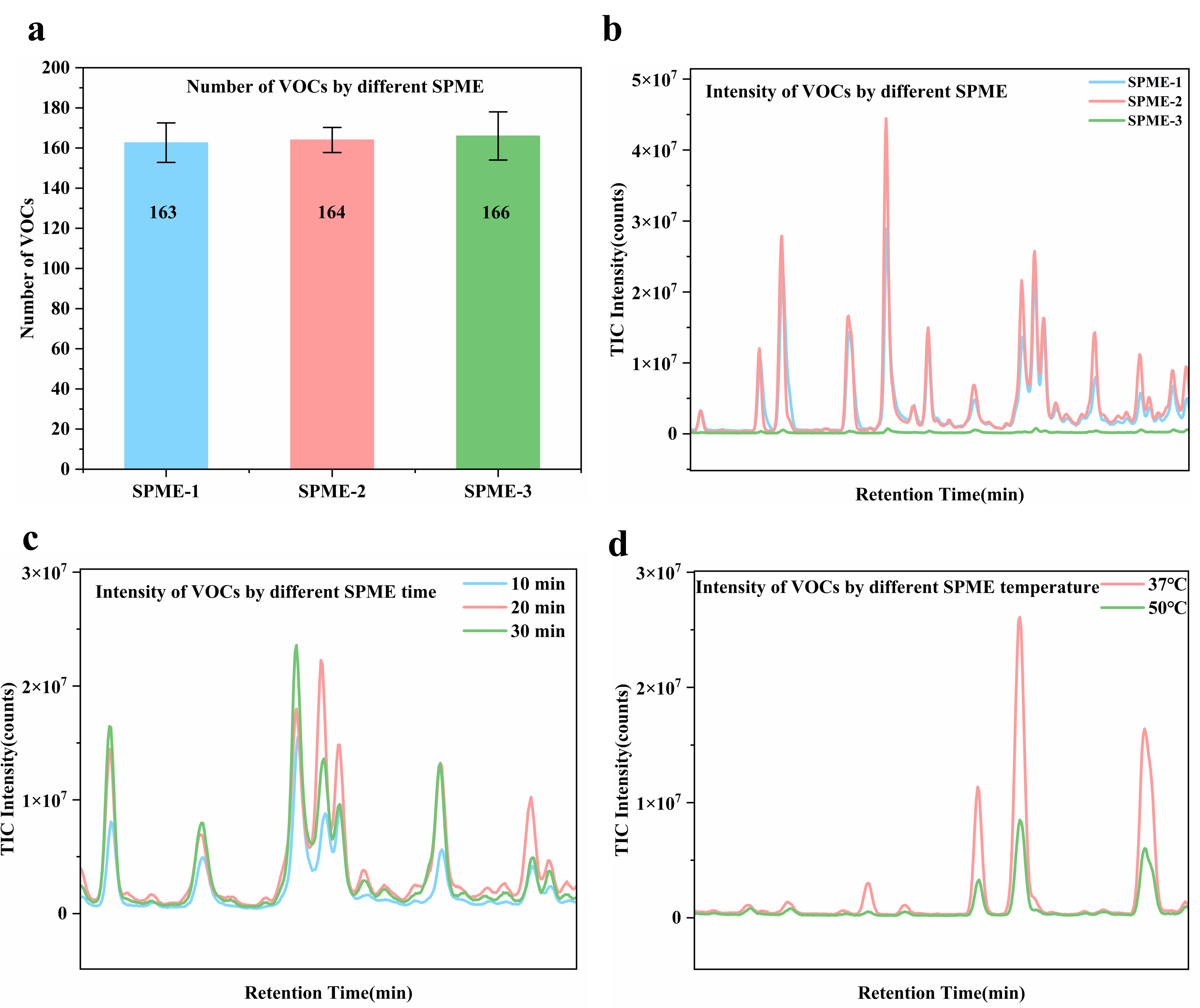


**Figure S2.** Optimization of SPME conditions: (a) The number of VOCs extracted by three SPME, （SPME-1: 50/30 μm DVB/CAR/PDMS, SPME-2: 65 μm PDMS/DVB, SPME-3: 100 μm PDMS）; (b) The intensity of VOCs extracted with three SPME; (c) The intensity of VOCs at three extraction time; (d) The intensity of VOCs at two extraction temperature.

**Figure S3.** (a) Cell morphology after detection at 37 °C; (b) Cell morphology after detection at 50 °C





**Figure S4.** (a) Stability analysis of the GC-MS instrument showing the relative fluctuation of intensity; (b) Peak area corresponding to different concentrations of ethanol.

**Results and Discusstion**

Based on the Mann-Whitney U-test analysis and Log_2_ FC a volcano plot (Fig. S5) was generated to visualize the changes in VOCs between the different cell lines. In resting state, inhibition by 2-DG, and inhibition by 3-BrPA, comparing BEAS-2B cells to A549 cells, we observed 26 / 13 / 29 VOCs that were downregulated and 9 / 6 / 19 VOCs that were upregulated. Similarly, in the BEAS-2B vs PC-9 comparison, 7 / 8 / 14 VOCs were downregulated, while 7 / 8 / 12 VOCs were upregulated. Finally, in the BEAS-2B vs NCI-H460 group, 29 / 28 / 29 VOCs were downregulated, and 17 / 8 / 9 VOCs were upregulated. To further refine our analysis, we applied OPLS-DA and considered VOCs with VIP > 1 and RSI > 800 in the NIST database. This selection process resulted in a subset of differential VOCs, which are summarized in Table 1, Table 2 and Table S1. Additionally, we provide Table S2 to illustrate the identification standard parts of these differential VOCs.


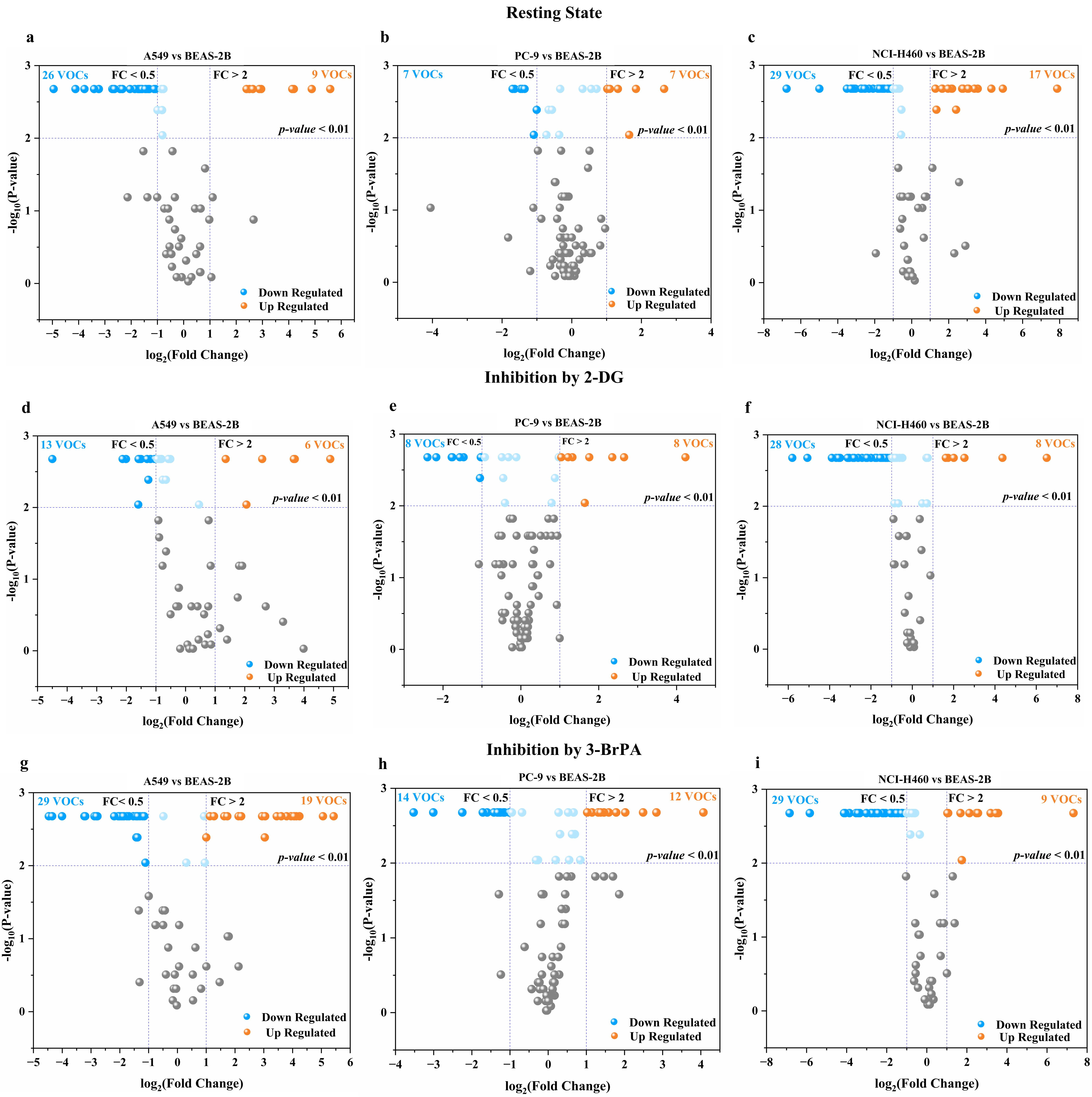


**Figure S5.** Volcano plots comparing the VOCs in headspace between Normal cell / Lung cancer cell, with significance based on p* < 0.01, FC < 0.5 or > 2.

**
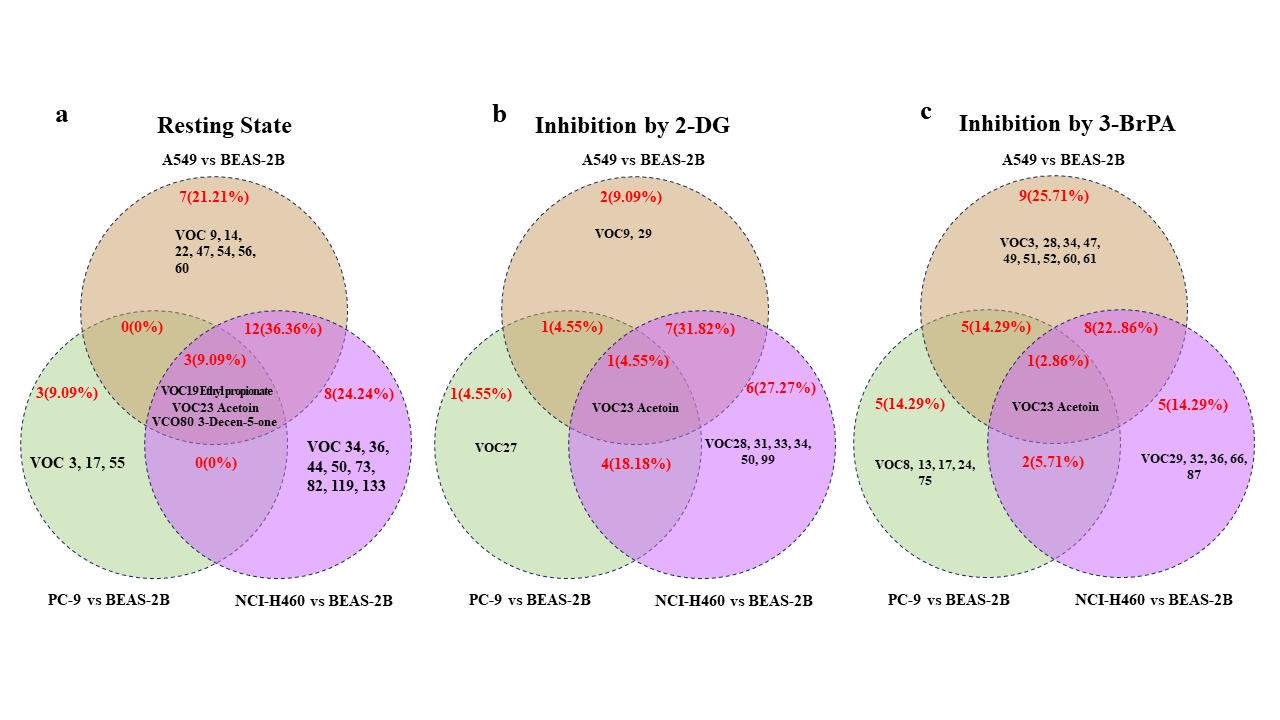
**

**Figure S6.** The Venn diagram of difference VOCs between three lung cancer cells and normal cells in resting state (a), 2-DG inhibited state (b), and 3-BrPA inhibited state, respectively. The brown circle represents A549 vs BEAS-2B, the green circle represents PC-9 vs BEAS-2B, and the purple circle represents NCI-H460 vs BEAS-2B. The red font indicates the number and percentage of different VOCs in each part, and the black font indicates the specific VOCs serial number or name.


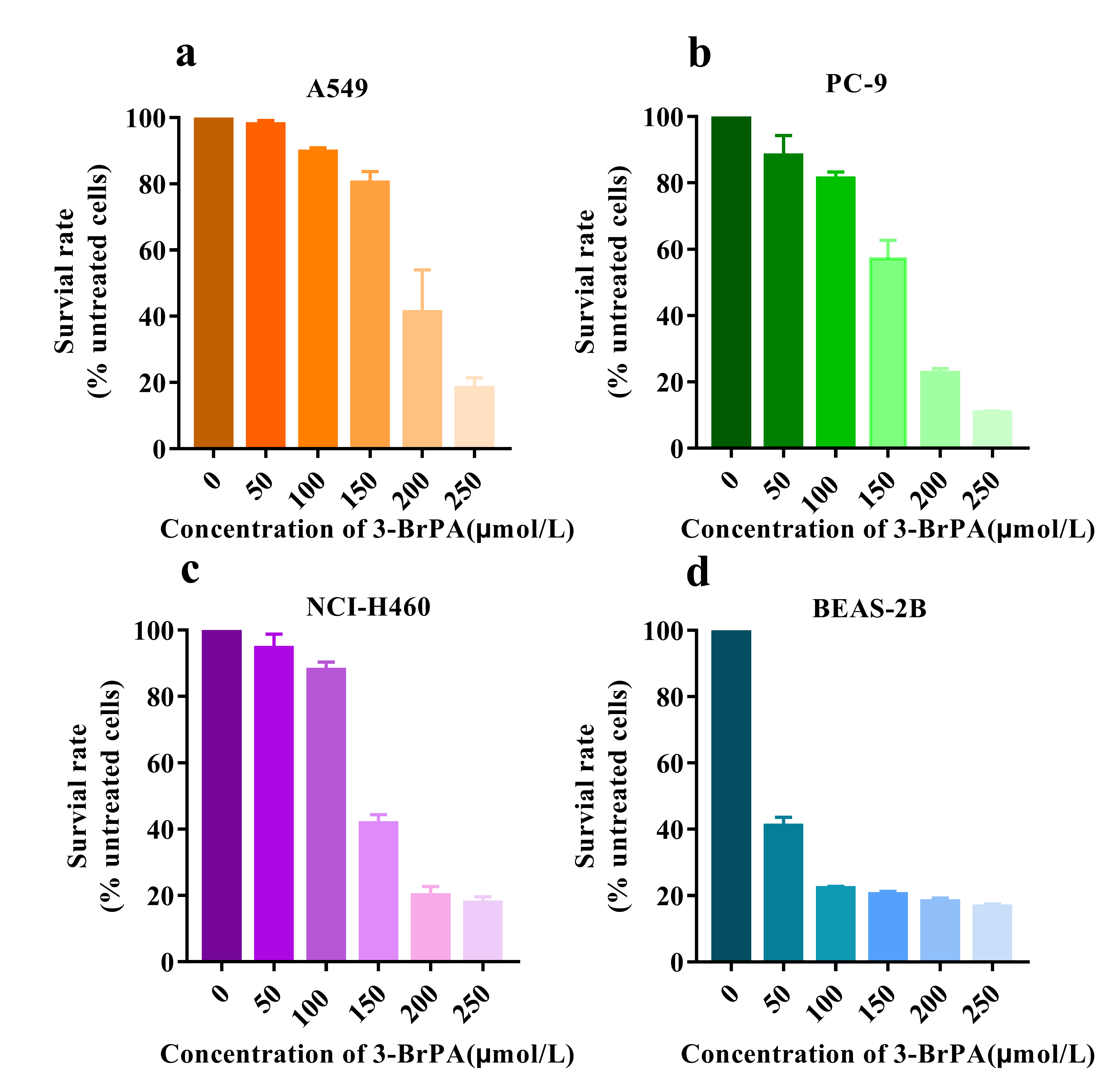


**Figure S7.** Survival rate of four cell types (A549, PC-9, NCI-H460, BEAS-2B) were assessed under varying concentrations of 3-BrPA.


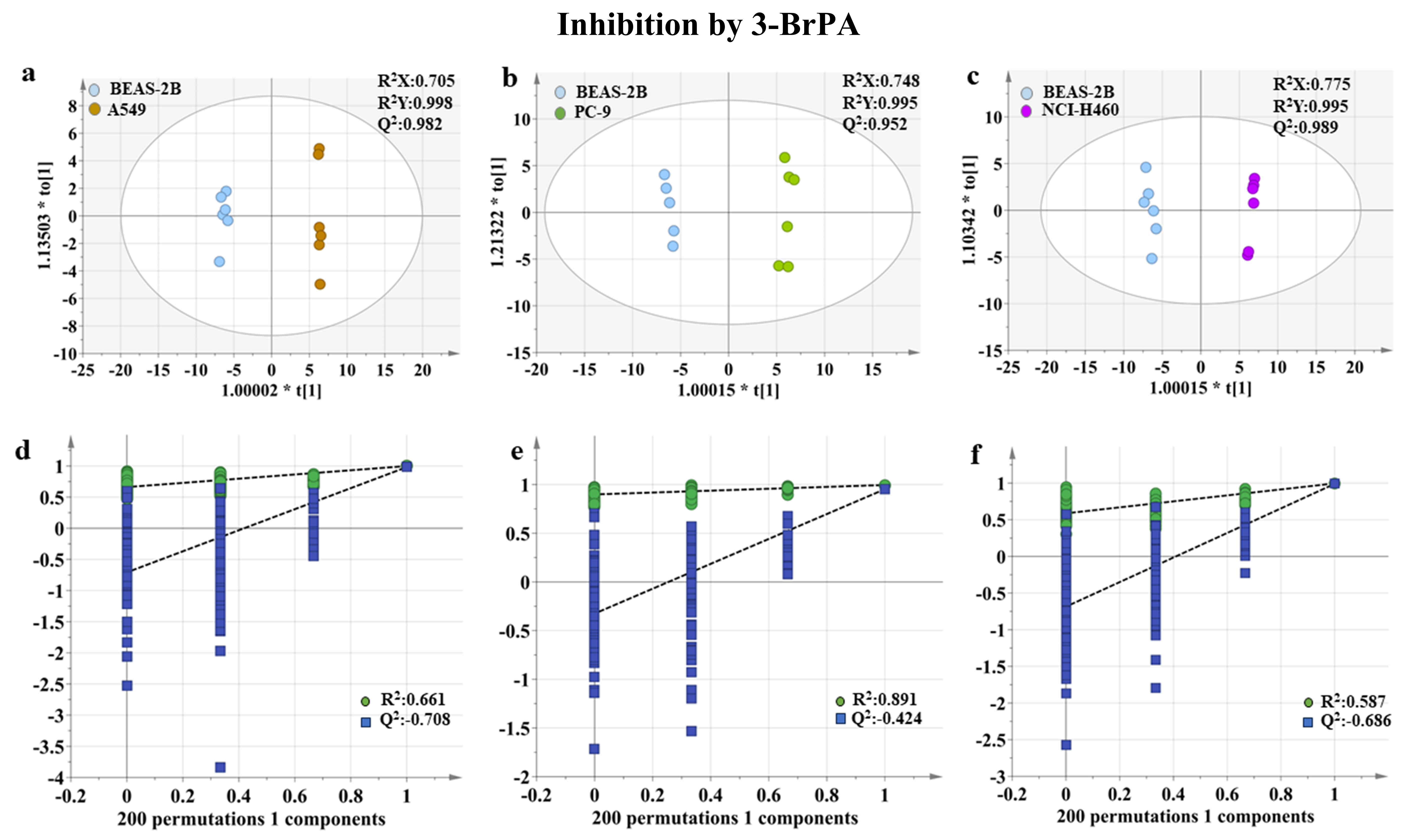


**Figure S8**. BEAS-2B and A549 (a) / PC-9 (b) / NCI-H460 (c) cells headspace VOCs OPLS-DA score plots after the inhibition by 3-BrPA (a, b, c), and the results of their corresponding 200 permutation tests (d, e, f).





**Figure** **S9.** Pathway of acetoin production^3,4^.

|  | Number | Name | HMDB ID | CAS | RT  (min) | RSI | RI^a^ | RI^b^ |
| --- | --- | --- | --- | --- | --- | --- | --- | --- |
| 1 | VOC3 | Ethanol* | HMDB0000108 | 64-17-5 | 1.78 | 905 | - | 481[5] |
| 2 | VOC4 | Acetone* | HMDB0001659 | 67-64-1 | 2.01 | 971 | - | 516[5] |
| 3 | VOC5 | Methylene chloride | HMDB0031548 | 75-09-2 | 2.35 | 958 | - | 516[6] |
| 4 | VOC8 | 2-Ethoxy-2-methyl-propane | - | 637-92-3 | 2.98 | 902 | 628 | 620[6] |
| 5 | VOC9 | 3,3,4,4-Tetrafluoro-hexane | - | 648-36-2 | 3.12 | 828 | 639 | - |
| 6 | VOC10 | Trichloromethane* | HMDB0029596 | 67-66-3 | 3.33 | 800 | 655 | 648[5] |
| 7 | VOC13 | 3-Methyl-butanal | - | 590-86-3 | 3.82 | 891 | 693 | 686[6] |
| 8 | VOC14 | Heptane* | HMDB0031447 | 142-82-5 | 3.93 | 854 | 700 | 700[6] |
| 9 | VOC15 | 1-Butanol* | HMDB0004327 | 71-36-3 | 4.08 | 911 | 710 | 688[6] |
| 10 | VOC17 | 5,5-Dimethyl-(Z)-2-hexene | - | 39761-61-0 | 4.23 | 877 | 719 | 721[6] |
| 11 | VOC19 | Ethyl propionate | HMDB0030058 | 105-37-3 | 4.45 | 881 | 731 | 716[5] |
| 12 | VOC22 | 2-Methyl-heptane | - | 592-27-8 | 4.98 | 925 | 762 | 762[6] |
| 13 | VOC23 | Acetoin* | HMDB0003243 | 513-86-0 | 5.14 | 812 | 772 | - |
| 14 | VOC24 | 3-Methyl-1-butanol* | HMDB0006007 | 123-51-3 | 5.25 | 880 | 778 | 788[5] |
| 15 | VOC28 | 2,4-Dimethyl-heptane | - | 2213-23-2 | 5.96 | 910 | 820 | 820[6] |
| 16 | VOC29 | Pyrrole | HMDB0035924 | 109-97-7 | 6.07 | 934 | 826 | 771[6] |
| 17 | VOC31 | 2,4-Dimethyl-1-heptene | - | 19549-87-2 | 6.39 | 862 | 845 | 846[6] |
| 18 | VOC32 | 3-Nonene | - | 20063-77-8 | 6.54 | 817 | 854 | - |
| 19 | VOC33 | 4-Methyl-octane | - | 2216-34-4 | 6.7 | 855 | 864 | 864[6] |
| 20 | VOC34 | 2-Methyl-ethyl ester butanoic acid | HMDB0033745 | 7452-79-1 | 6.85 | 861 | 873 | 870[6] |
| 21 | VOC36 | Ethylbenzene* | HMDB0059905 | 100-41-4 | 7.12 | 939 | 889 | 888[5] |
| 22 | VOC37 | p-Xylene* | HMDB0059924 | 106-42-3 | 7.24 | 934 | 896 | 896[5] |
| 23 | VOC44 | 3-Carene | HMDB0035619 | 13466-78-9 | 8.23 | 923 | 953 | 1046[5] |
| 24 | VOC47 | 2-Methyl-nonane | - | 871-83-0 | 8.46 | 890 | 966 | 965[6] |
| 25 | VOC49 | Oxime-, methoxy-phenyl- | - | NA | 9 | 829 | 996 | - |
| 26 | VOC50 | Decane* | HMDB0031450 | 124-18-5 | 9.07 | 933 | 1000 | 1000 |
| 27 | VOC51 | 2-Pentyl-furan | HMDB0013824 | 3777-69-3 | 9.22 | 831 | 1009 | 1001[6] |
| 28 | VOC52 | 2-5-Dimethyl-nonane | - | 17302-27-1 | 9.33 | 856 | 1015 | 1015[6] |
| 29 | VOC54 | 4-Ethyl-2,2,6,6-tetramethy1-heptane | - | 62108-31-0 | 9.51 | 832 | 1026 | - |
| 30 | VOC55 | 2,2,7,7-Tetramethyl-octane | - | 1071-31-4 | 9.71 | 859 | 1038 | 1036[6] |
| 31 | VOC56 | 2,3,6,7-Tetramethyl-octane | - | 52670-34-5 | 9.83 | 817 | 1045 | - |
| 32 | VOC59 | 5-Ethyl-2,2,3-trimethyl-heptane | - | 62199-06-8 | 10.2 | 899 | 1067 | - |
| 33 | VOC60 | 2,2,4,4-Tetramethyl-petane | - | 1070-87-7 | 10.24 | 893 | 1069 | - |
| 34 | VOC61 | 2-Ethyl-1-hexanol* | HMDB0031231 | 50373-29-0 | 10.33 | 930 | 1074 | 1078[7] |
| 35 | VOC66 | 2,6-Dimethyl-decane | - | 13150-81-7 | 10.83 | 868 | 1104 | 1105[6] |
| 36 | VOC73 | Dimethyl benzenemethanol | - | 617-94-7 | 11.65 | 870 | 1155 | 1102[6] |
| 37 | VOC75 | 2,6,10-Trimethyl-dodecane | - | 3891-98-3 | 11.88 | 853 | 1170 | - |
| 38 | VOC78 | Dodecane* | HMDB0031444 | 112-40-3 | 12.36 | 920 | 1200 | 1200 |
| 39 | VOC80 | 3-Decen-5-one | - | 32064-73-6 | 12.76 | 812 | 1225 | - |
| 40 | VOC82 | 2,6,10-Trimethyl-tetradecane | - | 14905-56-7 | 12.98 | 800 | 1240 | - |
| 41 | VOC87 | Benzene,1,3-bis(1,1-dimethylethyl)- | - | 1014-60-4 | 13.53 | 881 | 1276 | 1279[7] |
| 42 | VOC97 | 3,7-Dimethyl-undecane | - | 17301-29-0 | 14.33 | 825 | 1323 | - |
| 43 | VOC99 | 2-Undecanone | HMDB0033713 | 112-12-9 | 14.6 | 899 | 1338 | 1310[6] |
| 44 | VOC119 | Pentadecane* | HMDB0059886 | 629-62-9 | 17.45 | 874 | 1500 | 1500 |
| 45 | VOC133 | Hexadecane* | HMDB0033792 | 544-76-3 | 19.49 | 834 | 1700 | 1700 |

**Table S1.** Qualitative parameters of differential VOCs.

* Indicating that the VOC has been confirmed for retention time obtained by standard chemical reagents. The retention index (RI^a^) values are the present experimental results, and the referenced RI^b^ values are taken from the literatures ^5,7^ (by means of the DB-624UI column) or are the closest RI values from the PubChem database^6^ (with the semi-standard non-polar column).

*RT:* Retention time, *RSI:* Reverse Search Index. *RI:* Retention index.

| Differential VOCs | Number | Name | HMDB ID | CAS | RT (min) | VIP | FDR | Log2(FC) |
| --- | --- | --- | --- | --- | --- | --- | --- | --- |
| A549 vs BEAS-2B |  |  |  |  |  |  |  |  |
| 1 | VOC3 | Ethanol | HMDB0000108 | 64-17-5 | 1.78 | 1.25 | <0.01 | -3.23 |
| 2 | VOC4 | Acetone | HMDB0001659 | 67-64-1 | 2.01 | 1.31 | <0.01 | -1.43 |
| 3 | VOC5 | Methylene chloride | HMDB0031548 | 75-09-2 | 2.35 | 1.26 | <0.01 | -1.69 |
| 4 | VOC9 | 3,3,4,4-Tetrafluoro-hexane | - | 648-36-2 | 3.12 | 1.28 | <0.01 | -2.06 |
| 5 | VOC10 | Trichloromethane | HMDB0029596 | 67-66-3 | 3.33 | 1.03 | <0.01 | -1.42 |
| 6 | VOC14 | Heptane | HMDB0031447 | 142-82-5 | 3.93 | 1.30 | <0.01 | -4.46 |
| 7 | VOC19 | Ethyl propionate | HMDB0030058 | 105-37-3 | 4.45 | 1.22 | <0.01 | -1.30 |
| 8 | VOC23 | Acetoin | HMDB0003243 | 513-86-0 | 5.14 | 1.31 | <0.01 | 3.79 |
| 9 | VOC28 | 2,4-Dimethyl-heptane | - | 2213-23-2 | 5.96 | 1.30 | <0.01 | -4.01 |
| 10 | VOC31 | 2,4-Dimethyl-1-heptene | - | NA | 6.39 | 1.24 | <0.01 | -1.93 |
| 11 | VOC34 | 2-Methyl-ethyl ester butanoic acid | HMDB0033745 | 7452-79-1 | 6.85 | 1.17 | <0.01 | -1.21 |
| 12 | VOC47 | 2-Methyl-nonane | - | 871-83-0 | 8.46 | 1.10 | <0.01 | -2.19 |
| 13 | VOC49 | Oxime-, methoxy-phenyl- | - | NA | 9.00 | 1.30 | <0.01 | -3.20 |
| 14 | VOC51 | 2-Pentyl-furan | HMDB0013824 | 3777-69-3 | 9.22 | 1.32 | <0.01 | 1.63 |
| 15 | VOC52 | 2-5-Dimethyl-nonane | - | 17302-27-1 | 9.33 | 1.27 | <0.01 | -2.78 |
| 16 | VOC54 | 4-Ethyl-2.2.6.6-tetramethy1-heptane | - | 62108-31-0 | 9.51 | 1.23 | <0.01 | -1.68 |
| 17 | VOC55 | 2,2,7,7-Tetramethyl-octane | - | 1071-31-4 | 9.71 | 1.30 | <0.01 | -2.03 |
| 18 | VOC59 | 5-Ethyl-2,2,3-trimethyl-heptane | - | 62199-06-8 | 10.20 | 1.19 | <0.01 | -1.85 |
| 19 | VOC60 | 2,2,4,4-Tetramethyl-petane | - | 1070-87-7 | 10.24 | 1.12 | <0.01 | -1.75 |
| 20 | VOC61 | 2-Ethyl-1-hexanol | HMDB0031231 | NA | 10.33 | 1.32 | <0.01 | -4.35 |
| 21 | VOC78 | Dodecane | HMDB0031444 | 112-40-3 | 12.36 | 1.18 | <0.01 | -1.38 |
| 22 | VOC80 | 3-Decen-5-one | - | 32064-73-6 | 12.76 | 1.25 | <0.01 | -1.88 |
| 23 | VOC97 | 3,7-Dimethyl-undecane | - | 17301-29-0 | 14.33 | 1.24 | <0.01 | -1.15 |
| PC-9 vs BEAS-2B |  |  |  |  |  |  |  |  |
| 1 | VOC5 | Methylene chloride | HMDB0031548 | 75-09-2 | 2.35 | 1.46 | <0.01 | 1.15 |
| 2 | VOC8 | 2-Ethoxy-2-methyl propane | - | 637-92-3 | 2.98 | 1.39 | <0.01 | 2.02 |
| 3 | VOC13 | 3-Methyl-butanal | - | 590-86-3 | 3.82 | 1.40 | <0.01 | -3.52 |
| 4 | VOC14 | Heptane | HMDB0031447 | 142-82-5 | 3.93 | 1.41 | <0.01 | -2.25 |
| 5 | VOC15 | 1-Butanol | HMDB0004327 | 71-36-3 | 4.08 | 1.43 | <0.01 | -1.28 |
| 6 | VOC17 | 5,5-Dimethyl-(Z)-2-hexene | - | 107-39-1 | 4.23 | 1.06 | <0.01 | -3.01 |
| 7 | VOC19 | Ethyl propionate | - | 105-37-3 | 4.45 | 1.31 | <0.01 | -1.14 |
| 8 | VOC23 | Acetoin | HMDB0003243 | 513-86-0 | 5.14 | 1.45 | <0.01 | 4.07 |
| 9 | VOC24 | 3-Methyl-1-butanol | HMDB0006007 | 123-51-3 | 5.25 | 1.38 | <0.01 | 1.31 |
| 10 | VOC44 | 3-Carene | HMDB0035619 | 13466-78-9 | 8.23 | 1.14 | <0.01 | 1.02 |
| 11 | VOC55 | 2,2,7,7-Tetramethyl-octane | - | 1071-31-4 | 9.71 | 1.40 | <0.01 | 1.03 |
| 12 | VOC75 | 2,6,10-Trimethyl-dodecane | - | 3891-98-3 | 11.88 | 1.33 | <0.01 | 1.78 |
| 13 | VOC80 | 3-Decen-5-one | - | 32064-73-6 | 12.76 | 1.35 | <0.01 | -1.42 |
| NCI-H460 vs BEAS-2B |  |  |  |  |  |  |  |  |
| 1 | VOC4 | Acetone | HMDB0001659 | 67-64-1 | 2.01 | 1.17 | <0.01 | -1.61 |
| 2 | VOC9 | 3,3,4,4-Tetrafluoro-hexane | - | 648-36-2 | 3.12 | 1.09 | <0.01 | -1.02 |
| 3 | VOC10 | Trichloromethane | HMDB0029596 | 67-66-3 | 3.33 | 1.07 | <0.01 | -2.47 |
| 4 | VOC15 | 1-Butanol | HMDB0004327 | 71-36-3 | 4.08 | 1.13 | <0.01 | -1.03 |
| 5 | VOC23 | Acetoin | HMDB0003243 | 513-86-0 | 5.14 | 1.13 | <0.01 | 4.46 |
| 6 | VOC29 | Pyrrole | HMDB0035924 | 109-97-7 | 6.07 | 1.00 | <0.01 | 1.75 |
| 7 | VOC31 | 2,4-Dimethyl-1-heptene | - | NA | 6.39 | 1.18 | <0.01 | -3.86 |
| 8 | VOC32 | 3-Nonene | - | 20063-77-8 | 6.54 | 1.16 | <0.01 | -6.84 |
| 9 | VOC36 | Ethylbenzene | HMDB0059905 | 100-41-4 | 7.12 | 1.11 | <0.01 | -1.21 |
| 10 | VOC44 | 3-Carene | HMDB0035619 | 13466-78-9 | 8.23 | 1.10 | <0.01 | -2.16 |
| 11 | VOC54 | 4-Ethyl-2,2,6,6-tetramethy1-heptane | - | 62108-31-0 | 9.51 | 1.15 | <0.01 | -1.75 |
| 12 | VOC59 | 5-Ethyl-2,2,3-trimethyl-heptane | - | 62199-06-8 | 10.20 | 1.08 | <0.01 | -1.21 |
| 13 | VOC66 | 2,6-Dimethyl-decane | - | 13150-81-7 | 10.83 | 1.09 | <0.01 | -1.21 |
| 14 | VOC78 | Dodecane | HMDB0031444 | 112-40-3 | 12.36 | 1.10 | <0.01 | -1.42 |
| 15 | VOC87 | 1,3-Bis(1,1-dimethylethyl)-benzene | - | 1014-60-4 | 13.53 | 1.17 | <0.01 | -3.43 |
| 16 | VOC97 | 3,7-Dimethyl-undecane | - | 17301-29-0 | 14.33 | 1.14 | <0.01 | -1.26 |

**Table S2.** Differences in VOCs between lung cancer cells (A549, PC-9, NCI-H460) and normal cells (BEAS-2B) after the glycolysis inhibition by 3-BrPA.

**References**

1. Chu, Y. J. *et al*. Variable VOCs in plastic culture flasks and their potential impact on cell volatile biomarkers. *Anal Bioanal Chem.* **412**(22), 5397-5408 (2020).

2. Liu, Y. *et al.* HS−SPME−GC−MS untargeted analysis of normal rat organs Ex Vivo: differential VOC discrimination and fingerprint VOC identification. *Anal Chem.* **95**(30), 11375-11382 (2023).

3. Lopez, J. M., Thomas, B. & Rehbein H. Acetoin degradation in Bacillus subtilis by direct oxidative cleavage. *Eur J Biochem.* **57**(2), 425-430 (1975).

4. MetaCyc Pathway: (R)-acetoin biosynthesis I. Available online: http://vm-trypanocyc.toulouse.inra.fr/META/NEWIMAGE?type=PATHWAY&object=PWY-5938&detail-level=3. (Accessed on 20 Mar 2024).

5. Dossin E. *et al.* Prediction models of retention indices for increased confidence in structural elucidation during complex matrix analysis: application to gas chromatography coupled with high resolution mass spectrometry. *Anal Chem.* **88**(15),7539-47(2016).

6. PubChem database. Available online: https://pubchem.ncbi.nlm.nih.gov. (Accessed on 27 June 2024).

7. O'Callaghan T.F. *et al.* Influence of supplemental feed choice for pasture-based cows on the fatty acid and volatile profile of milk. *Foods.* **8**(4), 137 (2019).
